# Multi-allelic haplotype model based on genetic partition for genomic prediction and variance component estimation using SNP markers

**DOI:** 10.1186/s12863-015-0301-1

**Published:** 2015-12-18

**Authors:** Yang Da

**Affiliations:** Department of Animal Science, University of Minnesota, Saint Paul, MN USA

**Keywords:** Haplotype, Genomic selection, Variance component, Heritability, BLUP, REML

## Abstract

**Background:**

The amount of functional genomic information has been growing rapidly but remains largely unused in genomic selection. Genomic prediction and estimation using haplotypes in genome regions with functional elements such as all genes of the genome can be an approach to integrate functional and structural genomic information for genomic selection. Towards this goal, this article develops a new haplotype approach for genomic prediction and estimation.

**Results:**

A multi-allelic haplotype model treating each haplotype as an ‘allele’ was developed for genomic prediction and estimation based on the partition of a multi-allelic genotypic value into additive and dominance values. Each additive value is expressed as a function of h − 1 additive effects, where h = number of alleles or haplotypes, and each dominance value is expressed as a function of h(h − 1)/2 dominance effects. For a sample of q individuals, the limit number of effects is 2q − 1 for additive effects and is the number of heterozygous genotypes for dominance effects. Additive values are factorized as a product between the additive model matrix and the h − 1 additive effects, and dominance values are factorized as a product between the dominance model matrix and the h(h − 1)/2 dominance effects. Genomic additive relationship matrix is defined as a function of the haplotype model matrix for additive effects, and genomic dominance relationship matrix is defined as a function of the haplotype model matrix for dominance effects. Based on these results, a mixed model implementation for genomic prediction and variance component estimation that jointly use haplotypes and single markers is established, including two computing strategies for genomic prediction and variance component estimation with identical results.

**Conclusion:**

The multi-allelic genetic partition fills a theoretical gap in genetic partition by providing general formulations for partitioning multi-allelic genotypic values and provides a haplotype method based on the quantitative genetics model towards the utilization of functional and structural genomic information for genomic prediction and estimation.

## Background

Genomic best linear unbiased prediction (GBLUP) using genome-wide single nucleotide polymorphism (SNP) markers can utilize a wealth of theoretical results and computational strategies of best linear unbiased prediction (BLUP) [1] that has become a standard approach for genetic evaluation, with dairy cattle having the most widespread use of BLUP worldwide [2–5]. The implementation of GBLUP within the BLUP framework is made possible by a genomic relationship matrix that replaces the pedigree relationship matrix in BLUP [6]. With genomic relationship matrix established, genomic estimation of variance components can also readily use the method of restricted maximum likelihood estimation (REML) [7], to be referred to as GREML (genomic REML). Using a quantitative genetics model as the unifying model, genomic relationship matrix is formulated by equaling the covariance of genomic values between two individuals to the corresponding pedigree covariance [8, 9]. Previously defined genomic relationships based on standardization of SNP coding [6, 8, 10, 11] can be considered as special cases of this unifying approach [9]. The quantitative genetics model partitions a genotypic value as the summation of a common mean, breeding value and dominance deviation [12–18]. Using matrix notations, this partition can be expressed as: **g** = **1**μ + **a** + **d** = **1**μ + **W**_α_**α** + **W**_δ_**δ**, where μ = common mean, **1** = column vector of 1’s, **a** = breeding values (additive values), **d =** dominance deviations (dominance values), **α** = SNP additive effects, **δ** = SNP dominance effects, **W**_α_ = model matrix of **α** as a function of SNP allele frequencies, and **W**_δ_ = model matrix of **δ** as a function of SNP allele frequencies. With the factorization of **a** = **W**_α_**α** and **d** = **W**_δ_**δ**, genomic additive relationship is a function of **W**_α_**W**_α_ ' and genomic dominance relationship is a function of **W**_δ_**W**_δ_ ' [9]. This approach for defining genomic relationships was only available for bi-allelic loci. Although SNPs are bi-allelic loci, the issue of multi-allelic loci for genomic prediction and estimation arises if each haplotype is treated as an ‘allele’ and the haplotype block containing the haplotypes is treated as a ‘locus’. For a multi-allelic locus, the partition of a genotypic value into additive and dominance values (**g** = **1**μ + **a** + **d**) was available [17] and the multi-allelic factorization of **a** = **W**_α_**α** and **d** = **W**_δ_**δ** was available for three alleles [19]. However, general factorization formulations for an arbitrary number of alleles were unavailable, and a method using such multi-allelic haplotype model for genomic prediction and estimation was unavailable.

Haplotype analysis is advantageous over single-locus analysis for several reasons: a haplotype is a functional unit [20], a haplotype contains combined effects of tightly linked cis-acting causal variants [21, 22], a phenotype is affected by multiple causal loci with weak LD (LD = linkage disequilibrium) [23], or a genomic region is subjected to selection with stronger LD than genome regions unaffected by selection [24, 25]. Haplotype analysis has been widely used in genetic and genomic studies [22, 26–28]. Relatively limited studies were available on using haplotypes compared to the literature on using single SNPs for genomic prediction. Methods to define haplotype blocks for genomic prediction included a constant number of SNPs per SNP block [29, 30], fixed block length [31], or LD blocks [32]. Haplotype coding methods for genomic prediction and estimation included 2-1-0 copies of a haplotype in the two-haplotype genotype [30, 33], or maternal or paternal haplotype [29]. Haplotype mixed model methods based on the quantitative genetics model with multi-allelic factorization of additive and dominance values were unavailable for genomic prediction and estimation. Functional genomic information has been growing rapidly but remains largely unused in genomic selection. Simulation study showed that genomic prediction using causal mutations could substantially improve prediction accuracy [34], and using SNPs in transcriptional regions [35] or location specific priors based on QTL mapping results [36] improved prediction accuracy. Haplotype analysis can be a useful tool to account for joint allelic effects unaccounted for by single-SNP analysis and we have obtained encouraging preliminary results of using haplotype analysis of functional genomic information [37, 38].

The purpose of this article is to develop a quantitative genetics based multi-allelic haplotype model as an alternative method to single-SNP analysis towards the integration of functional and structural genomic information for genomic selection. This development includes deriving general multi-allelic partition of genotypic values with factorization for defining genomic relationships using haplotypes, and deriving mixed model formulations for genomic prediction and estimation that can use haplotypes separately or jointly with single SNPs.

## Methods

### Allelic mean and population mean of multi-allelic genotypic values

A set of m SNP markers are assumed available, and r haplotype blocks are defined from some of the m SNPs across the genome. Each haplotype block is treated as a ‘locus’ and each haplotype within the haplotype block is treated as an ‘allele’. Each locus (haplotype block) is assumed to have h alleles (haplotypes) denoted by *A*_i_, …, *A*_h_, with allele frequency of p_i_ for *A*_i_, i = 1, …, h, and ∑_i = 1_^h^p_i_ = 1. The allelic array in the population is ∑_i = 1_^h^p_i_*A*_i_. Let P_ij_ = frequency of *A*_i_*A*_j_ genotype, ∑_i = 1_^h^∑_j = 1_^h^P_ij_*A*_i_*A*_j_ = the genotypic array of the population, and g_ij_ = genotypic value of *A*_i_*A*_j_ genotype, i,j = 1,…,h. Hardy-Weinberg equilibrium (HWE) is assumed so that the genotypic array of the population is the squared allelic array, i.e., ∑_i = 1_^h^∑_j = 1_^h^P_ij_*A*_i_*A*_j_ = (∑_i = 1_^h^p_i_*A*_i_)^2^. Allele frequency of *A*_i_ is calculated as:

1 $$ {\mathrm{p}}_{\mathrm{i}}={\mathrm{P}}_{\mathrm{i}\mathrm{i}}+\frac{1}{2}{\displaystyle {\sum}_{\begin{array}{l}\mathrm{j}=1\\ {}\mathrm{j}\ne \mathrm{i}\end{array}}^{\mathrm{h}}{\mathrm{P}}_{\mathrm{i}\mathrm{j}}} $$

The allelic mean of *A*_i_ allele is the weighted mean of all genotypic values with the *A*_i_ allele, with each genotypic value weighted by the number of copies of the *A*_i_ allele the genotype carries. The general expression of the allelic mean without requiring HWE is a conditional mean [13] and simplifies to a weighted average of genotypic values with allele frequencies as the weights under the HWE assumption [13, 17], i.e.,

2 $$ {\upmu}_{\mathrm{i}}=\left[2{\mathrm{P}}_{\mathrm{i}\mathrm{i}}{\mathrm{g}}_{\mathrm{i}\mathrm{i}}+{\displaystyle {\sum}_{\mathrm{j}\ne \mathrm{i}}^{\mathrm{h}}{\mathrm{P}}_{\mathrm{i}\mathrm{j}}{\mathrm{g}}_{\mathrm{i}\mathrm{j}}}\right]/\left[2{\mathrm{P}}_{\mathrm{i}\mathrm{i}}+{\displaystyle {\sum}_{\mathrm{j}\ne \mathrm{i}}^{\mathrm{h}}{\mathrm{P}}_{\mathrm{i}\mathrm{j}}}\right]={\displaystyle {\sum}_{\mathrm{j}=1}^{\mathrm{h}}{\mathrm{p}}_{\mathrm{j}}{\mathrm{g}}_{\mathrm{i}\mathrm{j}}} $$

The population mean is the mean of all genotypic values in the population. The general formula without requiring HWE and its expression as a weighted average of allelic means with allele frequencies as the weights requiring HWE are:

3 $$ \upmu ={\displaystyle {\sum}_{\mathrm{i}=1}^{\mathrm{h}}{\displaystyle {\sum}_{\mathrm{j}=1}^{\mathrm{h}}{\mathrm{P}}_{\mathrm{i}\mathrm{j}}{\mathrm{g}}_{\mathrm{i}\mathrm{j}}}}={\displaystyle {\sum}_{\mathrm{i}=1}^{\mathrm{h}}{\mathrm{p}}_{\mathrm{i}}^2{\mathrm{g}}_{\mathrm{i}\mathrm{i}}+2{\displaystyle {\sum}_{\mathrm{i}=1}^{\mathrm{h}-1}{\displaystyle {\sum}_{\mathrm{j}=\mathrm{i}+1}^{\mathrm{h}}{\mathrm{p}}_{\mathrm{i}}{\mathrm{p}}_{\mathrm{j}}{\mathrm{g}}_{\mathrm{i}\mathrm{j}}}}}={\displaystyle {\sum}_{\mathrm{k}=1}^{\mathrm{h}}{\mathrm{p}}_{\mathrm{k}}{\upmu}_{\mathrm{k}}} $$

The expressions of μ_i_ = ∑_j = 1_^h^p_j_g_ij_ and μ = ∑_k = 1_^h^p_k_μ_k_ play an important role in the derivations to factorize additive and dominance values and in defining fundamental genetic parameters of quantitative traits.

### Multi-allelic effect, additive effect, additive value

The allelic effect (average effect) of allele *A*_i_ (i = 1,…h) is the deviation of the allelic mean from the population mean. From Eqs. 2 and 3, the allelic effect of *A*_i_ is:

4 $$ {\mathrm{a}}_{\mathrm{i}}={\upmu}_{\mathrm{i}}-\upmu ={\displaystyle {\sum}_{\mathrm{j}\ne \mathrm{i}}^{\mathrm{h}}{\mathrm{p}}_{\mathrm{j}}\left({\upmu}_{\mathrm{i}}-{\upmu}_{\mathrm{j}}\right)}={\displaystyle {\sum}_{\mathrm{j}\ne \mathrm{i}}^{\mathrm{h}}{\mathrm{p}}_{\mathrm{j}}{\upalpha}_{\mathrm{i}\mathrm{j}}} $$

where α_ij_ is the additive effect or the average effect of gene substitution that is the difference between the allelic effects of the two alleles defined by Eq. 4, i.e.,

5 $$ {\upalpha}_{\mathrm{i}\mathrm{j}}={\mathrm{a}}_{\mathrm{i}}-{\mathrm{a}}_{\mathrm{j}}={\upmu}_{\mathrm{i}}-{\upmu}_{\mathrm{j}}={\displaystyle {\sum}_{\mathrm{k}=1}^{\mathrm{h}}{\mathrm{p}}_{\mathrm{k}}}\left({\mathrm{g}}_{\mathrm{i}\mathrm{k}}-{\mathrm{g}}_{\mathrm{j}\mathrm{k}}\right)=-\upalpha \mathrm{j}\mathrm{i} $$

For h alleles, h(h − 1)/2 α_ij_ parameters of Eq. 5 are possible but these parameters are not independent for all ij values. An example of this dependency is:

6 $$ {\upalpha}_{\mathrm{ij}}={\upalpha}_{1\mathrm{j}}-\upalpha 1\mathrm{i} $$

Based on Eq. 6, h-1 independent additive effects can be defined:

7 $$ {\upalpha}_{1\mathrm{k}}={\mathrm{a}}_1-{\mathrm{a}}_{\mathrm{k}}={\upmu}_1-{\upmu}_{\mathrm{k}},\mathrm{k}=2,\dots \mathrm{h} $$

where μ_1_ = allelic mean of allele 1 that is used as the reference allele (e.g., defining the most frequent allele as ‘allele 1’). It is readily seen that α_ii_ = 0. The derivation process will allow the presence of α_ii_ but the final results will be based on the h−1 independent additive effects of α_lk_ defined by Eq. 7. All the h(h − 1)/2 possible α_ij_ parameters can be expressed in terms of the h−1 independent α_lk_ parameters through Eq. 6. The additive value (breeding value) of genotype *A*_i_*A*_j_ is the summation of the two allelic effects of the genotype, i.e.,

8 $$ {\mathrm{a}}_{\mathrm{i}\mathrm{j}}={\mathrm{a}}_{\mathrm{i}}+{\mathrm{a}}_{\mathrm{j}} $$

Each additive value defined by Eq. 8 will be shown to be a function of all h−1 additive effects defined by Eq. 7.

### Dominance effect and dominance value

Dominance effect of *A*_i_*A*_j_ genotype (δ_ij_) is the deviation of the heterozygous genotypic value from the average of the two homozygous genotypic values, i.e.,

9 $$ {\updelta}_{\mathrm{ij}}={\mathrm{g}}_{\mathrm{ij}}-\frac{1}{2}\left({\mathrm{g}}_{\mathrm{ii}}+{\mathrm{g}}_{\mathrm{jj}}\right) $$

With the above definition, dominance effect is the unique effect of a heterozygous genotype. Therefore, the number of dominance effects is the same as number of heterozygous genotypes, and the maximum number of dominance effects is h(h − 1)/2. It is readily seen from Eq. 9 that δ_ii_ = 0. The derivation process will allow the presence of δ_ii_ but the final results will not have δ_ii_. Dominance value or dominance deviation is the deviation of the genotypic value from the common mean and additive value, i.e.,

10 $$ {\mathrm{d}}_{\mathrm{ij}}={\mathrm{g}}_{\mathrm{ij}}-\upmu -{\mathrm{a}}_{\mathrm{ij}} $$

An important difference between ‘dominance value’ and ‘dominance effect’ is that a homozygous genotype may have non-zero dominance value but always has zero dominance effect. Each dominance value defined by Eq. 10 will be shown to be a function of all h(h − 1)/2 dominance effects defined by Eq. 9.

### Multi-allelic partition of genotypic value and variance

The genotypic value of a multi-allelic genotype has the same partition as for a bi-allelic locus [17], i.e.,

11 $$ {\mathrm{g}}_{\mathrm{ij}}=\upmu +{\mathrm{a}}_{\mathrm{ij}}+{\mathrm{d}}_{\mathrm{ij}} $$

with E(a_ij_) = 0 and E(d_ij_) = 0. The multi-allelic genotypic variance (σ_g_^2^) also has the same partition as for a bi-allelic locus [17], i.e., σ_g_^2^ = σ_a_^2^ + σ_d_^2^, where σ_a_^2^ = additive variance, and σ_d_^2^ = dominance variance. The multi-allelic haplotype model to be developed starts with the factorization of the additive and dominance values in Eq. 11.

## Results and discussion

### Factorization of additive and dominance values

From Eqs. 4–7, an allelic effect can be expressed as:

12 $$ {\mathrm{a}}_{\mathrm{i}}={\upmu}_{\mathrm{i}}-\upmu ={\displaystyle {\sum}_{\mathrm{k}\ne \mathrm{i}}^{\mathrm{h}}{\mathrm{p}}_{\mathrm{k}}{\upalpha}_{\mathrm{i}\mathrm{k}}}={\displaystyle {\sum}_{\mathrm{k}\ne \mathrm{i}}^{\mathrm{h}}{\mathrm{p}}_{\mathrm{k}}\left({\upalpha}_{1\mathrm{k}}-\upalpha 1\mathrm{i}\right)}=-\left(1-{\mathrm{p}}_{\mathrm{i}}\right)\upalpha +1\mathrm{i}{\displaystyle {\sum}_{\mathrm{k}\ne \mathrm{i}}^{\mathrm{h}}{\mathrm{p}}_{\mathrm{k}}{\upalpha}_{1\mathrm{k}}} $$

where α_lk_ is defined by Eq. 7. Equation 12 shows that an allelic effect is a function of all h-1 parameters of additive effects denoted by α_lk_. The additive values (breeding values) of *A*_*i*_*A*_*j*_ and *A*_*i*_*A*_*i*_ genotypes can be expressed as:

13 $$ \begin{array}{l}{\mathrm{a}}_{\mathrm{i}\mathrm{j}}={\mathrm{a}}_{\mathrm{i}}+{\mathrm{a}}_{\mathrm{j}}=\left[-\left(1-{\mathrm{p}}_{\mathrm{i}}\right)\upalpha +1\mathrm{i}{\displaystyle {\sum}_{\mathrm{k}\ne \mathrm{i}}^{\mathrm{h}}{\mathrm{p}}_{\mathrm{k}}{\upalpha}_{1\mathrm{k}}}\right]+\left[-\left(1-{\mathrm{p}}_{\mathrm{j}}\right)\upalpha +1\mathrm{j}{\displaystyle {\sum}_{\mathrm{k}\ne \mathrm{i}}^{\mathrm{h}}{\mathrm{p}}_{\mathrm{k}}{\upalpha}_{1\mathrm{k}}}\right]\\ {}=-\left(1-2{\mathrm{p}}_{\mathrm{i}}\right)\upalpha -1\mathrm{i}\left(1-2{\mathrm{p}}_{\mathrm{j}}\right)\upalpha +1\mathrm{j}2{\displaystyle {\sum}_{\mathrm{k}\ne \mathrm{i}\mathrm{j}}^{\mathrm{h}}{\mathrm{p}}_{\mathrm{k}}{\upalpha}_{1\mathrm{k}}}\end{array} $$

14 $$ {\mathrm{a}}_{\mathrm{i}\mathrm{i}}=2{\mathrm{a}}_{\mathrm{i}}=-2\left(1-2{\mathrm{p}}_{\mathrm{i}}\right)\upalpha +1\mathrm{i}2{\displaystyle {\sum}_{\mathrm{k}\ne \mathrm{i}\mathrm{j}}^{\mathrm{h}}{\mathrm{p}}_{\mathrm{k}}{\upalpha}_{1\mathrm{k}}} $$

In Eqs. 13 and 14, α_li_ = 0 if i = 1 and α_1j_ = 0 if j = 1. From Eqs. 1–3 and 9–10, the dominance value of the *A*_i_*A*_j_ genotype can be expressed as

15 $$ \begin{array}{l}{\mathrm{d}}_{\mathrm{i}\mathrm{j}}={\mathrm{g}}_{\mathrm{i}\mathrm{j}}-\upmu -{\mathrm{a}}_{\mathrm{i}}-{\mathrm{a}}_{\mathrm{j}}={\mathrm{g}}_{\mathrm{i}\mathrm{j}}-{\upmu}_{\mathrm{i}}-{\upmu}_{\mathrm{j}}+\upmu = \left({\mathrm{g}}_{\mathrm{i}\mathrm{j}}-{\upmu}_{\mathrm{i}}\right)-\left({\upmu}_{\mathrm{j}}-\upmu \right)\\ {}={\displaystyle {\sum}_{\mathrm{k}\ne \mathrm{j}}^{\mathrm{h}}{\mathrm{p}}_{\mathrm{k}}\left({\mathrm{g}}_{\mathrm{i}\mathrm{j}}-{\mathrm{g}}_{\mathrm{i}\mathrm{k}}\right)}-{\displaystyle {\sum}_{\mathrm{k}\ne \mathrm{j}}^{\mathrm{h}}{\mathrm{p}}_{\mathrm{k}}\left({\upmu}_{\mathrm{j}}-{\upmu}_{\mathrm{k}}\right)} = {\displaystyle {\sum}_{\mathrm{k}\ne \mathrm{j}}^{\mathrm{h}}{\mathrm{p}}_{\mathrm{k}}\left[\left({\mathrm{g}}_{\mathrm{i}\mathrm{j}}-{\upmu}_{\mathrm{j}}\right)-\left({\mathrm{g}}_{\mathrm{i}\mathrm{k}}-{\upmu}_{\mathrm{k}}\right)\right]}\\ {}={\displaystyle {\sum}_{\mathrm{k}\ne \mathrm{j}}^{\mathrm{h}}{\mathrm{p}}_{\mathrm{k}}\left[{\displaystyle {\sum}_{\mathrm{f}\ne \mathrm{i}}^{\mathrm{h}}{\mathrm{p}}_{\mathrm{f}}\left({\mathrm{g}}_{\mathrm{i}\mathrm{j}}-{\mathrm{g}}_{\mathrm{j}\mathrm{f}}\right)}-{\displaystyle {\sum}_{\mathrm{f}\ne \mathrm{i}}^{\mathrm{h}}{\mathrm{p}}_{\mathrm{f}}\left({\mathrm{g}}_{\mathrm{i}\mathrm{k}}-{\mathrm{g}}_{\mathrm{k}\mathrm{f}}\right)}\right]}\\ {}={\displaystyle {\sum}_{\mathrm{k}\ne \mathrm{j}}^{\mathrm{h}}{\mathrm{p}}_{\mathrm{k}}{\displaystyle {\sum}_{\mathrm{f}\ne \mathrm{i}}^{\mathrm{h}}{\mathrm{p}}_{\mathrm{f}}\left({\mathrm{g}}_{\mathrm{i}\mathrm{j}}-{\mathrm{g}}_{\mathrm{i}\mathrm{k}}-{\mathrm{g}}_{\mathrm{j}\mathrm{f}}+{\mathrm{g}}_{\mathrm{k}\mathrm{f}}\right)}}\end{array} $$

In Eq. 15, the quantity g_ij_ − g_ik_ − g_jf_ + g_kf_ has two positive terms and two negative terms, and each subscript is associated with a positive term and a negative term. Using this fact and the definition of dominance effect (δ_ij_) of Eq. 9 with δ_ii_ = 0, g_ij_ − g_ik_ − g_jf_ + g_kf_ can be expressed as:

16 $$ {\mathrm{g}}_{\mathrm{ij}}-{\mathrm{g}}_{\mathrm{ik}}-{\mathrm{g}}_{\mathrm{jf}}+{\mathrm{g}}_{\mathrm{kf}}={\updelta}_{\mathrm{ij}}-{\updelta}_{\mathrm{ik}}-{\updelta}_{\mathrm{jf}}+{\updelta}_{\mathrm{kf}} $$

Combining Eqs. 15 and 16 with Eq. 10 and using p_j_ = 1 − ∑_k ≠ j_^h^p_k_ (Eq. 1) yields:

17 $$ \begin{array}{l}{\mathrm{d}}_{\mathrm{i}\mathrm{j}}={\displaystyle {\sum}_{\mathrm{k}\ne \mathrm{j}}^{\mathrm{h}}{\mathrm{p}}_{\mathrm{k}}{\displaystyle {\sum}_{\mathrm{f}\ne \mathrm{i}}^{\mathrm{h}}{\mathrm{p}}_{\mathrm{f}}\left({\updelta}_{\mathrm{i}\mathrm{j}}-{\updelta}_{\mathrm{i}\mathrm{k}}-{\updelta}_{\mathrm{j}\mathrm{f}}+{\updelta}_{\mathrm{k}\mathrm{f}}\right)}}\\ {}={\displaystyle {\sum}_{\mathrm{k}\ne \mathrm{j}}^{\mathrm{h}}{\mathrm{p}}_{\mathrm{k}}\left[{\displaystyle {\sum}_{\mathrm{f}\ne \mathrm{i}}^{\mathrm{h}}{\mathrm{p}}_{\mathrm{f}}\left({\updelta}_{\mathrm{i}\mathrm{j}}-{\updelta}_{\mathrm{i}\mathrm{k}}\right)}-{\displaystyle {\sum}_{\mathrm{f}\ne \mathrm{i}}^{\mathrm{h}}{\mathrm{p}}_{\mathrm{f}}\left({\updelta}_{\mathrm{j}\mathrm{f}}-{\updelta}_{\mathrm{k}\mathrm{f}}\right)}\right]}\\ {}={\displaystyle {\sum}_{\mathrm{k}\ne \mathrm{j}}^{\mathrm{h}}{\mathrm{p}}_{\mathrm{k}}\left[\left(1-{\mathrm{p}}_{\mathrm{i}}\right)\left({\updelta}_{\mathrm{i}\mathrm{j}}-{\updelta}_{\mathrm{i}\mathrm{k}}\right)-{\displaystyle {\sum}_{\mathrm{f}\ne \mathrm{i}}^{\mathrm{h}}{\mathrm{p}}_{\mathrm{f}}{\updelta}_{\mathrm{j}\mathrm{f}}}+{\displaystyle {\sum}_{\mathrm{f}\ne \mathrm{i}}^{\mathrm{h}}{\mathrm{p}}_{\mathrm{f}}}{\updelta}_{\mathrm{k}\mathrm{f}}\right]}\\ {}=\left(1-{\mathrm{p}}_{\mathrm{i}}\right)\left(1-{\mathrm{p}}_{\mathrm{j}}\right){\updelta}_{\mathrm{i}\mathrm{j}}-\left(1-{\mathrm{p}}_{\mathrm{i}}\right){\displaystyle {\sum}_{\mathrm{k}\ne \mathrm{j}}^{\mathrm{h}}{\mathrm{p}}_{\mathrm{k}}{\updelta}_{\mathrm{i}\mathrm{k}}-{\displaystyle {\sum}_{\mathrm{k}\ne \mathrm{j}}^{\mathrm{h}}{\mathrm{p}}_{\mathrm{k}}\left({\displaystyle {\sum}_{\mathrm{f}\ne \mathrm{i}}^{\mathrm{h}}{\mathrm{p}}_{\mathrm{f}}{\updelta}_{\mathrm{j}\mathrm{f}}}-{\displaystyle {\sum}_{\mathrm{f}\ne \mathrm{i}}^{\mathrm{h}}{\mathrm{p}}_{\mathrm{f}}}{\updelta}_{\mathrm{k}\mathrm{f}}\right)}}\\ {}=\left(1-{\mathrm{p}}_{\mathrm{i}}\right)\left(1-{\mathrm{p}}_{\mathrm{j}}\right){\updelta}_{\mathrm{i}\mathrm{j}}-\left(1-{\mathrm{p}}_{\mathrm{i}}\right){\displaystyle {\sum}_{\mathrm{k}\ne \mathrm{j}}^{\mathrm{h}}{\mathrm{p}}_{\mathrm{k}}{\updelta}_{\mathrm{i}\mathrm{k}}-\left(1-{\mathrm{p}}_{\mathrm{j}}\right){\displaystyle {\sum}_{\mathrm{f}\ne \mathrm{i}}^{\mathrm{h}}{\mathrm{p}}_{\mathrm{f}}{\updelta}_{\mathrm{j}\mathrm{f}}}+{\displaystyle {\sum}_{\mathrm{k}\ne \mathrm{j}}^{\mathrm{h}}{\mathrm{p}}_{\mathrm{k}}}{\displaystyle {\sum}_{\mathrm{f}\ne \mathrm{i}}^{\mathrm{h}}{\mathrm{p}}_{\mathrm{f}}}{\updelta}_{\mathrm{k}\mathrm{f}}}\end{array} $$

In Eq. 17,

18 $$ \begin{array}{l}{\displaystyle {\sum}_{\mathrm{k}\ne \mathrm{j}}^{\mathrm{h}}{\mathrm{p}}_{\mathrm{k}}}{\displaystyle {\sum}_{\mathrm{f}\ne \mathrm{i}}^{\mathrm{h}}{\mathrm{p}}_{\mathrm{f}}}{\updelta}_{\mathrm{k}\mathrm{f}}={\mathrm{p}}_{\mathrm{i}}{\mathrm{p}}_{\mathrm{j}}{\updelta}_{\mathrm{i}\mathrm{j}}+{\mathrm{p}}_{\mathrm{i}}{\displaystyle {\sum}_{\mathrm{f}\ne \mathrm{i},\mathrm{k}}^{\mathrm{h}}{\mathrm{p}}_{\mathrm{f}}}{\updelta}_{\mathrm{j}\mathrm{f}}+{\mathrm{p}}_{\mathrm{j}}{\displaystyle {\sum}_{\mathrm{k}\ne \mathrm{j},\mathrm{f}}^{\mathrm{h}}{\mathrm{p}}_{\mathrm{k}}}{\updelta}_{\mathrm{j}\mathrm{k}}+{\displaystyle {\sum}_{\mathrm{k}\ne \mathrm{i},\mathrm{j}}^{\mathrm{h}}{\mathrm{p}}_{\mathrm{k}}}{\displaystyle {\sum}_{\mathrm{f}\ne \mathrm{k}}^{\mathrm{h}}{\mathrm{p}}_{\mathrm{f}}}{\updelta}_{\mathrm{k}\mathrm{f}}\\ {}={\mathrm{p}}_{\mathrm{i}}{\mathrm{p}}_{\mathrm{j}}{\updelta}_{\mathrm{i}\mathrm{j}}+{\mathrm{p}}_{\mathrm{i}}{\displaystyle {\sum}_{\mathrm{k}\ne \mathrm{i},\mathrm{j}}^{\mathrm{h}}{\mathrm{p}}_{\mathrm{k}}}{\updelta}_{\mathrm{i}\mathrm{k}}+{\mathrm{p}}_{\mathrm{j}}{\displaystyle {\sum}_{\mathrm{f}\ne \mathrm{i},\mathrm{j}}^{\mathrm{h}}{\mathrm{p}}_{\mathrm{f}}}{\updelta}_{\mathrm{j}\mathrm{f}}+2{\displaystyle {\sum}_{\mathrm{k}\ne \mathrm{i},\mathrm{j}}^{\mathrm{h}-1}{\mathrm{p}}_{\mathrm{k}}}{\displaystyle {\sum}_{\mathrm{f}=\mathrm{k}+1}^{\mathrm{h}}{\mathrm{p}}_{\mathrm{f}}}{\updelta}_{\mathrm{k}\mathrm{f}}\end{array} $$

Combining Eqs. 17 and 18 yields:

19 $$ \begin{array}{l}{\mathrm{d}}_{\mathrm{i}\mathrm{j}}={\mathrm{g}}_{\mathrm{i}\mathrm{j}}-\upmu -{\mathrm{a}}_{\mathrm{i}}-{\mathrm{a}}_{\mathrm{j}}=\left[1-{\mathrm{p}}_{\mathrm{i}}\left(1-{\mathrm{p}}_{\mathrm{j}}\right)-{\mathrm{p}}_{\mathrm{j}}\left(1-{\mathrm{p}}_{\mathrm{i}}\right)\right]{\updelta}_{\mathrm{i}\mathrm{j}}\\ {}\kern0.84em -\left(1-2{\mathrm{p}}_{\mathrm{i}}\right){\displaystyle {\sum}_{\mathrm{k}\ne \mathrm{i},\mathrm{j}}^{\mathrm{h}}{\mathrm{p}}_{\mathrm{k}}{\updelta}_{\mathrm{i}\mathrm{k}}-\left(1-2{\mathrm{p}}_{\mathrm{j}}\right){\displaystyle {\sum}_{\mathrm{f}\ne \mathrm{i},\mathrm{j}}^{\mathrm{h}}{\mathrm{p}}_{\mathrm{f}}{\updelta}_{\mathrm{j}\mathrm{f}}}}+2{\displaystyle {\sum}_{\mathrm{k}\ne \mathrm{i},\mathrm{j}}^{\mathrm{h}-1}{\mathrm{p}}_{\mathrm{k}}}{\displaystyle {\sum}_{\mathrm{f}=\mathrm{k}+1}^{\mathrm{h}}{\mathrm{p}}_{\mathrm{f}}}{\updelta}_{\mathrm{k}\mathrm{f}}\end{array} $$

20 $$ {\mathrm{d}}_{\mathrm{i}\mathrm{i}}={\mathrm{g}}_{\mathrm{i}\mathrm{i}}-\upmu -2{\mathrm{a}}_{\mathrm{i}}=-2\left(1-{\mathrm{p}}_{\mathrm{i}}\right){\displaystyle {\sum}_{\mathrm{k}\ne \mathrm{i}}^{\mathrm{h}}{\mathrm{p}}_{\mathrm{k}}{\updelta}_{\mathrm{i}\mathrm{k}}}+2{\displaystyle {\sum}_{\mathrm{k}\ne \mathrm{i}}^{\mathrm{h}-1}{\mathrm{p}}_{\mathrm{k}}}{\displaystyle {\sum}_{\mathrm{f}=\mathrm{k}+1}^{\mathrm{h}}{\mathrm{p}}_{\mathrm{f}}}{\updelta}_{\mathrm{k}\mathrm{f}} $$

Equations 13 and 14 show that each additive value is a function of all h − 1 additive effects defined by Eq. 7, and Eqs. 19–20 show that each dominance value is a function of all h(h − 1)/2 dominance effects defined by Eq. 9. Equations 13 and 14 provide the additive coding and Eqs. 19 and 20 provide the dominance coding of each multi-allelic genotype for the mixed model implementation.

### Multi-allelic haplotype model based on multi-allelic genetic partition

Using the results of factorization of additive and dominance values given by Eqs. 13–14 and 19–20, the multi-allelic haplotype model treating each haplotype as an ‘allele’ by Eq. 11 can be expressed as:

21 $$ {\mathrm{g}}_{\mathrm{ij}}=\upmu +{\mathrm{a}}_{\mathrm{ij}}+{\mathrm{d}}_{\mathrm{ij}}=\upmu +{\displaystyle {\sum}_{\mathrm{k}=2}^{\mathrm{h}}{\mathrm{w}}_{\upalpha}^{\mathrm{ij},\mathrm{k}}{\upalpha}_{1\mathrm{k}}}+{\displaystyle {\sum}_{\mathrm{k}=1}^{\mathrm{h}-1}{\displaystyle {\sum}_{\mathrm{f}=\mathrm{k}+1}^{\mathrm{h}}{\mathrm{w}}_{\updelta}^{\mathrm{ij},\mathrm{k}\mathrm{f}}{\updelta}_{\mathrm{k}\mathrm{f}}}} $$

In w_α_^ij,k^, superscripts ij are for the genotype of *A*_i_*A*_j_ and superscript k is for α_lk_. In w_δ_^ij,kf^, superscripts ij are for d_ij_ and superscripts kf are for δ_kf_. From Eqs. 13 and 14, the additive coding (w_α_^ij,k^) of a multi-allelic genotype is:

22 $$ {\mathrm{w}}_{\upalpha}^{\mathrm{ij},\mathrm{k}}=2{\mathrm{p}}_{\mathrm{k}}\kern0.5em \mathrm{f}\mathrm{o}\mathrm{r}\kern0.5em \mathrm{i},\mathrm{j}\ne \mathrm{k}\kern0.1em \left({\mathrm{a}}_{\mathrm{ij}}\kern0.5em \mathrm{and}\kern0.5em {\upalpha}_{1\mathrm{k}}\kern0.5em \mathrm{do}\kern0.5em \mathrm{not}\kern0.5em \mathrm{share}\kern0.5em \mathrm{allele}\kern0.5em \mathrm{k}\right) $$

23 $$ {\mathrm{w}}_{\upalpha}^{\mathrm{ij},\mathrm{k}}=-\left(1-2{\mathrm{p}}_{\mathrm{k}}\right)\kern0.5em \mathrm{f}\mathrm{o}\mathrm{r}\kern0.5em \mathrm{i}\ne \mathrm{j}\kern0.5em \mathrm{but}\kern0.5em \mathrm{i}=\mathrm{k}\kern0.5em \mathrm{o}\mathrm{r}\kern0.5em \mathrm{j}=\mathrm{k}\left({\mathrm{a}}_{\mathrm{ij}}\kern0.5em \mathrm{and}\kern0.5em {\upalpha}_{1\mathrm{k}}\kern0.5em \mathrm{share}\kern0.5em \mathrm{allele}\kern0.5em \mathrm{k},\mathrm{i}\ne \mathrm{k}\right) $$

24 $$ {\mathrm{w}}_{\upalpha}^{\mathrm{ij},\mathrm{k}}=-2\left(1-{\mathrm{p}}_{\mathrm{k}}\;\right)\ \mathrm{f}\mathrm{o}\mathrm{r}\ \mathrm{i}=\mathrm{j}=\mathrm{k}\ \left({\mathrm{a}}_{\mathrm{ij}}\kern0.5em \mathrm{and}\kern0.5em {\upalpha}_{1\mathrm{k}}\kern0.5em \mathrm{share}\ \mathrm{allele}\ \mathrm{k},\ \mathrm{i}=\mathrm{j}\right) $$

From Eqs. 19 and 20, the dominance coding (w_δ_^ij,kf^) of a multi-allelic genotype is:

25 $$ {\mathrm{w}}_{\updelta}^{\mathrm{i}\mathrm{j},\mathrm{k}\mathrm{f}}=1-{\mathrm{p}}_{\mathrm{i}}\left(1-{\mathrm{p}}_{\mathrm{j}}\right)-{\mathrm{p}}_{\mathrm{j}}\left(1-{\mathrm{p}}_{\mathrm{i}}\right)\kern0.37em \mathrm{f}\mathrm{o}\mathrm{r}\ \mathrm{i}\mathrm{j}=\mathrm{k}\mathrm{f}\ \left({\mathrm{d}}_{\mathrm{i}\mathrm{j}}\kern0.5em \mathrm{and}\kern0.5em {\updelta}_{\mathrm{kf}}\kern0.5em \mathrm{share}\ 2\ \mathrm{alleles}\right) $$

26 $$ {\mathrm{w}}_{\updelta}^{\mathrm{i}\mathrm{j},\mathrm{k}\mathrm{f}}=-{\mathrm{p}}_{\mathrm{k}}\left(1-2{\mathrm{p}}_{\mathrm{i}}\right)\ \mathrm{f}\mathrm{o}\mathrm{r}\ \mathrm{i}\ne \mathrm{j}\ \mathrm{and}\ \mathrm{i}=\mathrm{f}\ \left({\mathrm{d}}_{\mathrm{i}\mathrm{j}}\kern0.5em \mathrm{and}\kern0.5em {\updelta}_{\mathrm{k}\mathrm{f}}\kern0.5em \mathrm{share}\ \mathrm{allele}\ \mathrm{f},\ \mathrm{i}\ne \mathrm{j}\right) $$

27 $$ {\mathrm{w}}_{\updelta}^{\mathrm{ij},\mathrm{k}\mathrm{f}}=-{\mathrm{p}}_{\mathrm{f}}\left(1-2{\mathrm{p}}_{\mathrm{j}}\right)\ \mathrm{f}\mathrm{o}\mathrm{r}\ \mathrm{i}\ne \mathrm{j}\ \mathrm{and}\ \mathrm{j}=\mathrm{k}\ \left({\mathrm{d}}_{\mathrm{ij}}\kern0.5em \mathrm{and}\kern0.5em {\updelta}_{\mathrm{kf}}\kern0.5em \mathrm{share}\ \mathrm{allele}\ \mathrm{k},\ \mathrm{i}\ne \mathrm{j}\right) $$

28 $$ {\mathrm{w}}_{\updelta}^{\mathrm{i}\mathrm{j},\mathrm{k}\mathrm{f}}=-2{\mathrm{p}}_{\mathrm{k}}\left(1-{\mathrm{p}}_{\mathrm{i}}\right)\kern0.37em \mathrm{f}\mathrm{o}\mathrm{r}\ \mathrm{i}=\mathrm{j}\ \mathrm{and}\ \mathrm{i}=\mathrm{f}\ \left({\mathrm{d}}_{\mathrm{i}\mathrm{j}}\kern0.5em \mathrm{and}\kern0.5em {\updelta}_{\mathrm{k}\mathrm{f}}\kern0.5em \mathrm{share}\ \mathrm{allele}\ \mathrm{f},\ \mathrm{i}=\mathrm{j}\right) $$

29 $$ {\mathrm{w}}_{\updelta}^{\mathrm{ij},\mathrm{k}\mathrm{f}}=2{\mathrm{p}}_{\mathrm{k}}{\mathrm{p}}_{\mathrm{f}}\kern0.37em \mathrm{f}\mathrm{o}\mathrm{r}\ \mathrm{i},\mathrm{j}\ne \mathrm{k},\mathrm{f}\ \left({\mathrm{d}}_{\mathrm{ij}}\kern0.5em \mathrm{and}\kern0.5em {\updelta}_{\mathrm{k}\mathrm{f}}\kern0.5em \mathrm{share}\ \mathrm{no}\ \mathrm{allele},\ \mathrm{i}=\mathrm{j}\ \mathrm{o}\mathrm{r}\ \mathrm{i}\ne \mathrm{j}\right) $$

For convenience of computer programming, Eqs. 22–24 can be characterized by whether a_ij_ and α_lk_ share no common allele (Eq. 22), or 1 common allele when i ≠ j (Eq. 23) or 1 common allele when i = j (Eq. 24). Similarly, between d_ij_ and δ_kf_, Eq. 25 shares two common alleles, Eqs. 26 and 27 share 1 common allele with i ≠ j, Eq. 28 shares one common allele with i = j, and Eq. 29 share no common allele. In Eqs. 25–29, p_i_ or p_j_ is the allele frequency of the shared allele between d_ij_ and δ_kf_ and p_k_ or p_f_ is the allele frequency of the non-shared allele between d_ij_ and δ_kf_. From Eqs. 21–29, the multi-allelic haplotype model for h(h + 1)/2 possible genotypic values (**g**) of a given haplotype block with h haplotypes can be expressed as:

30 $$ \mathbf{g}=\mathbf{1}\upmu +{\mathbf{a}}_{\mathrm{h}}+{\mathbf{d}}_{\mathrm{h}}=\mathbf{1}\upmu +{\mathbf{W}}_{\upalpha \mathrm{h}}{\upalpha}_{\mathrm{h}}+{\mathbf{W}}_{\updelta \mathrm{h}}{\updelta}_{\mathrm{h}} $$

where μ = common mean, **1** = [h(h + 1)/2] × 1 column vector of 1’s, **a**_h_ = **W**_αh_**α**_h_ = [h(h + 1)/2] × 1 column vector of additive values (breeding values), **d**_h_ = **W**_δh_**δ**_h_ = [h(h + 1)/2] × 1 column vector of dominance values (dominance deviations), **W**_αh_ = [h(h + 1)/2] × (h − 1) model matrix of **α**_h_with w_α_^ij,k^ defined by Eqs. 22–24, d_h_ = [h(h + 1)/2] × 1 column vector of dominance values (dominance deviations), **W**_δh_ = [h(h + 1)/2] × [h(h − 1)/2] matrix of **δ**_h_ with w_δ_^ij,kf^ defined by Eqs. 25–29, and **α**_h_ = (h − 1) × 1 column vector with α_lk_ defined by Eq. 7, and **δ**_h_ = [h(h − 1)/2] × 1 column vector with δ_kf_ defined by Eq. 9.

### Numerical example of multi-allelic genetic partition

A hypothetical numerical example is used to illustrate the genetic partition of multi-allelic genotypic values described by Eqs. 21–30. Four haplotypes as ‘alleles’ are assumed with frequencies in Table 1 and genotypic values in Table 2. The common mean of the genotypic values using Eq. 3 is: μ = 22.09. The additive effects of the four haplotypes defined by Eqs. 5–7, are:

**Table 1:** Four hypothetical haplotypes and their frequencies (h = 4)

Haplotype	1	2	3	4
Frequency	0.4	0.3	0.2	0.1

**Table 2:** Genotypic values of haplotype genotypes (g_*ij*_ = g_*ji*_)

Haplotype	1	2	3	4
1	g_11_ = 25	g_12_ = 18	g_13_ = 15	g_14_ = 10
2		g_22_ = 30	g_23_ = 33	g_24_ = 40
3			g_33_ = 17	g_34_ = 12
4				g_44_ = 35

$$ {\boldsymbol{\upalpha}}_{\mathrm{h}}\hbox{'}=\left[\begin{array}{ccc}\hfill -7.4\hfill & \hfill -1.1\hfill & \hfill -2.5\hfill \end{array}\right]\hbox{'}, $$

and the dominance effects defined by Eq. 9 are:

$$ {\boldsymbol{\updelta}}_{\mathrm{h}}\hbox{'}=\left[\begin{array}{cccccc}\hfill -9.5\hfill & \hfill -6\hfill & \hfill -20\hfill & \hfill 9.5\hfill & \hfill 7.5\hfill & \hfill -14\hfill \end{array}\right]\hbox{'}. $$

Using Eqs. 13–14 and 22–24, the additive values (breeding values) are:

$$ {\mathbf{a}}_{\mathrm{h}}=\left[\begin{array}{c}\hfill {\mathrm{a}}_{11}\hfill \\ {}\hfill {\mathrm{a}}_{22}\hfill \\ {}\hfill {\mathrm{a}}_{33}\hfill \\ {}\hfill {\mathrm{a}}_{44}\hfill \\ {}\hfill {\mathrm{a}}_{12}\hfill \\ {}\hfill {\mathrm{a}}_{13}\hfill \\ {}\hfill {\mathrm{a}}_{14}\hfill \\ {}\hfill {\mathrm{a}}_{23}\hfill \\ {}\hfill {\mathrm{a}}_{24}\hfill \\ {}\hfill {\mathrm{a}}_{34}\hfill \end{array}\right]=\left[\begin{array}{ccc}\hfill 2{\mathrm{p}}_2\hfill & \hfill 2{\mathrm{p}}_3\hfill & \hfill 2{\mathrm{p}}_4\hfill \\ {}\hfill -2\left(1-{\mathrm{p}}_2\right)\hfill & \hfill 2{\mathrm{p}}_3\hfill & \hfill 2{\mathrm{p}}_4\hfill \\ {}\hfill 2{\mathrm{p}}_2\hfill & \hfill -2\left(1-{\mathrm{p}}_3\right)\hfill & \hfill 2{\mathrm{p}}_4\hfill \\ {}\hfill 2{\mathrm{p}}_2\hfill & \hfill 2{\mathrm{p}}_3\hfill & \hfill -2\left(1-{\mathrm{p}}_4\right)\hfill \\ {}\hfill -\left(1-2{\mathrm{p}}_2\right)\hfill & \hfill 2{\mathrm{p}}_3\hfill & \hfill 2{\mathrm{p}}_4\hfill \\ {}\hfill 2{\mathrm{p}}_2\hfill & \hfill -\left(1-2{\mathrm{p}}_3\right)\hfill & \hfill 2{\mathrm{p}}_4\hfill \\ {}\hfill 2{\mathrm{p}}_2\hfill & \hfill 2{\mathrm{p}}_3\hfill & \hfill -\left(1-2{\mathrm{p}}_4\right)\hfill \\ {}\hfill -\left(1-2{\mathrm{p}}_2\right)\hfill & \hfill -\left(1-2{\mathrm{p}}_3\right)\hfill & \hfill 2{\mathrm{p}}_4\hfill \\ {}\hfill -\left(1-2{\mathrm{p}}_2\right)\hfill & \hfill 2{\mathrm{p}}_3\hfill & \hfill -\left(1-2{\mathrm{p}}_4\right)\hfill \\ {}\hfill 2{\mathrm{p}}_2\hfill & \hfill -\left(1-2{\mathrm{p}}_3\right)\hfill & \hfill -\left(1-2{\mathrm{p}}_4\right)\hfill \end{array}\right]\left[\begin{array}{c}\hfill {\alpha}_{12}\hfill \\ {}\hfill {\alpha}_{13}\hfill \\ {}\hfill {\alpha}_{14}\hfill \end{array}\right]=\left[\begin{array}{ccc}\hfill 0.6\hfill & \hfill 0.4\hfill & \hfill 0.2\hfill \\ {}\hfill -1.4\hfill & \hfill 0.4\hfill & \hfill 0.2\hfill \\ {}\hfill 0.6\hfill & \hfill -1.6\hfill & \hfill 0.2\hfill \\ {}\hfill 0.6\hfill & \hfill 0.4\hfill & \hfill -1.8\hfill \\ {}\hfill -0.4\hfill & \hfill 0.4\hfill & \hfill 0.2\hfill \\ {}\hfill 0.6\hfill & \hfill -0.6\hfill & \hfill 0.2\hfill \\ {}\hfill 0.6\hfill & \hfill 0.4\hfill & \hfill -0.8\hfill \\ {}\hfill -0.4\hfill & \hfill -0.6\hfill & \hfill 0.2\hfill \\ {}\hfill -0.4\hfill & \hfill 0.4\hfill & \hfill -0.8\hfill \\ {}\hfill 0.6\hfill & \hfill -0.6\hfill & \hfill -0.8\hfill \end{array}\right]\left[\begin{array}{c}\hfill -7.4\hfill \\ {}\hfill -1.1\hfill \\ {}\hfill -2.5\hfill \end{array}\right]=\left[\begin{array}{c}\hfill -5.38\hfill \\ {}\hfill 9.42\hfill \\ {}\hfill -3.18\hfill \\ {}\hfill -0.38\hfill \\ {}\hfill 2.02\hfill \\ {}\hfill -4.28\hfill \\ {}\hfill -2.88\hfill \\ {}\hfill 3.12\hfill \\ {}\hfill 4.52\hfill \\ {}\hfill -1.78\hfill \end{array}\right]. $$

Using Eqs. 19–20 and 25–29, the dominance values (dominance deviations) are:

$$ \begin{array}{l}{\mathbf{d}}_{\mathrm{h}}=\left[\begin{array}{c}\hfill {\mathrm{d}}_{11}\hfill \\ {}\hfill {\mathrm{d}}_{22}\hfill \\ {}\hfill {\mathrm{d}}_{33}\hfill \\ {}\hfill {\mathrm{d}}_{44}\hfill \\ {}\hfill {\mathrm{d}}_{12}\hfill \\ {}\hfill {\mathrm{d}}_{13}\hfill \\ {}\hfill {\mathrm{d}}_{14}\hfill \\ {}\hfill {\mathrm{d}}_{23}\hfill \\ {}\hfill {\mathrm{d}}_{24}\hfill \\ {}\hfill {\mathrm{d}}_{34}\hfill \end{array}\right]=\left[\begin{array}{cccccc}\hfill -2{\mathrm{p}}_2\left(1-{\mathrm{p}}_1\right)\hfill & \hfill -2{\mathrm{p}}_3\left(1-{\mathrm{p}}_1\right)\hfill & \hfill -2{\mathrm{p}}_4\left(1-{\mathrm{p}}_1\right)\hfill & \hfill 2{\mathrm{p}}_2{\mathrm{p}}_3\hfill & \hfill 2{\mathrm{p}}_2{\mathrm{p}}_4\hfill & \hfill 2{\mathrm{p}}_3{\mathrm{p}}_4\hfill \\ {}\hfill -2{\mathrm{p}}_1\left(1-{\mathrm{p}}_2\right)\hfill & \hfill 2{\mathrm{p}}_1{\mathrm{p}}_3\hfill & \hfill 2{\mathrm{p}}_1{\mathrm{p}}_4\hfill & \hfill -2{\mathrm{p}}_3\left(1-{\mathrm{p}}_2\right)\hfill & \hfill -2{\mathrm{p}}_4\left(1-{\mathrm{p}}_2\right)\hfill & \hfill 2{\mathrm{p}}_3{\mathrm{p}}_4\hfill \\ {}\hfill 2{\mathrm{p}}_1{\mathrm{p}}_2\hfill & \hfill -2{\mathrm{p}}_1\left(1-{\mathrm{p}}_3\right)\hfill & \hfill 2{\mathrm{p}}_1{\mathrm{p}}_4\hfill & \hfill -2{\mathrm{p}}_2\left(1-{\mathrm{p}}_3\right)\hfill & \hfill 2{\mathrm{p}}_2{\mathrm{p}}_4\hfill & \hfill -2{\mathrm{p}}_4\left(1-{\mathrm{p}}_3\right)\hfill \\ {}\hfill 2{\mathrm{p}}_1{\mathrm{p}}_2\hfill & \hfill 2{\mathrm{p}}_1{\mathrm{p}}_3\hfill & \hfill -2{\mathrm{p}}_1\left(1-{\mathrm{p}}_4\right)\hfill & \hfill 2{\mathrm{p}}_2{\mathrm{p}}_3\hfill & \hfill -2{\mathrm{p}}_2\left(1-{\mathrm{p}}_4\right)\hfill & \hfill -2{\mathrm{p}}_3\left(1-{\mathrm{p}}_4\right)\hfill \\ {}\hfill {\mathrm{w}}_{\updelta}^{12,12}\hfill & \hfill -{\mathrm{p}}_3\left(1-2{\mathrm{p}}_1\right)\hfill & \hfill -{\mathrm{p}}_4\left(1-2{\mathrm{p}}_1\right)\hfill & \hfill -{\mathrm{p}}_3\left(1-2{\mathrm{p}}_2\right)\hfill & \hfill -{\mathrm{p}}_4\left(1-2{\mathrm{p}}_2\right)\hfill & \hfill 2{\mathrm{p}}_3{\mathrm{p}}_4\hfill \\ {}\hfill -{\mathrm{p}}_2\left(1-2{\mathrm{p}}_1\right)\hfill & \hfill {\mathrm{w}}_{\updelta}^{13,13}\hfill & \hfill -{\mathrm{p}}_4\left(1-2{\mathrm{p}}_1\right)\hfill & \hfill -{\mathrm{p}}_2\left(1-2{\mathrm{p}}_3\right)\hfill & \hfill 2{\mathrm{p}}_2{\mathrm{p}}_4\hfill & \hfill -{\mathrm{p}}_4\left(1-2{\mathrm{p}}_3\right)\hfill \\ {}\hfill -{\mathrm{p}}_2\left(1-2{\mathrm{p}}_1\right)\hfill & \hfill -{\mathrm{p}}_3\left(1-2{\mathrm{p}}_1\right)\hfill & \hfill {\mathrm{w}}_{\updelta}^{14,14}\hfill & \hfill 2{\mathrm{p}}_2{\mathrm{p}}_3\hfill & \hfill -{\mathrm{p}}_2\left(1-2{\mathrm{p}}_4\right)\hfill & \hfill -{\mathrm{p}}_3\left(1-2{\mathrm{p}}_4\right)\hfill \\ {}\hfill -{\mathrm{p}}_1\left(1-2{\mathrm{p}}_2\right)\hfill & \hfill -{\mathrm{p}}_1\left(1-2{\mathrm{p}}_3\right)\hfill & \hfill 2{\mathrm{p}}_1{\mathrm{p}}_4\hfill & \hfill {\mathrm{w}}_{\updelta}^{23,23}\hfill & \hfill -{\mathrm{p}}_4\left(1-2{\mathrm{p}}_2\right)\hfill & \hfill -{\mathrm{p}}_4\left(1-2{\mathrm{p}}_3\right)\hfill \\ {}\hfill -{\mathrm{p}}_1\left(1-2{\mathrm{p}}_2\right)\hfill & \hfill 2{\mathrm{p}}_1{\mathrm{p}}_3\hfill & \hfill -{\mathrm{p}}_1\left(1-2{\mathrm{p}}_4\right)\hfill & \hfill -{\mathrm{p}}_3\left(1-2{\mathrm{p}}_2\right)\hfill & \hfill {\mathrm{w}}_{\updelta}^{24,24}\hfill & \hfill -{\mathrm{p}}_3\left(1-2{\mathrm{p}}_4\right)\hfill \\ {}\hfill 2{\mathrm{p}}_1{\mathrm{p}}_2\hfill & \hfill -{\mathrm{p}}_1\left(1-2{\mathrm{p}}_3\right)\hfill & \hfill -{\mathrm{p}}_1\left(1-2{\mathrm{p}}_4\right)\hfill & \hfill -{\mathrm{p}}_2\left(1-2{\mathrm{p}}_3\right)\hfill & \hfill -{\mathrm{p}}_2\left(1-2{\mathrm{p}}_4\right)\hfill & \hfill {\mathrm{w}}_{\updelta}^{34,34}\hfill \end{array}\right]\left[\begin{array}{c}\hfill {\updelta}_{12}\hfill \\ {}\hfill {\updelta}_{13}\hfill \\ {}\hfill {\updelta}_{14}\hfill \\ {}\hfill {\updelta}_{23}\hfill \\ {}\hfill {\updelta}_{24}\hfill \\ {}\hfill {\updelta}_{34}\hfill \end{array}\right]\\ {}\kern2em =\left[\begin{array}{cccccc}\hfill -0.36\hfill & \hfill -0.24\hfill & \hfill -0.12\hfill & \hfill 0.12\hfill & \hfill 0.06\hfill & \hfill 0.04\hfill \\ {}\hfill -0.56\hfill & \hfill 0.16\hfill & \hfill 0.08\hfill & \hfill -0.28\hfill & \hfill -0.14\hfill & \hfill 0.04\hfill \\ {}\hfill 0.24\hfill & \hfill -0.64\hfill & \hfill 0.08\hfill & \hfill -0.48\hfill & \hfill 0.06\hfill & \hfill -0.16\hfill \\ {}\hfill 0.24\hfill & \hfill 0.16\hfill & \hfill -0.72\hfill & \hfill 0.12\hfill & \hfill -0.54\hfill & \hfill -0.36\hfill \\ {}\hfill 0.54\hfill & \hfill -0.04\hfill & \hfill -0.02\hfill & \hfill -0.08\hfill & \hfill -0.04\hfill & \hfill 0.04\hfill \\ {}\hfill -0.06\hfill & \hfill 0.56\hfill & \hfill -0.02\hfill & \hfill -0.18\hfill & \hfill 0.06\hfill & \hfill -0.06\hfill \\ {}\hfill -0.06\hfill & \hfill -0.04\hfill & \hfill 0.58\hfill & \hfill 0.12\hfill & \hfill -0.24\hfill & \hfill -0.16\hfill \\ {}\hfill -0.16\hfill & \hfill -0.24\hfill & \hfill 0.08\hfill & \hfill 0.62\hfill & \hfill -0.04\hfill & \hfill -0.06\hfill \\ {}\hfill -0.16\hfill & \hfill 0.16\hfill & \hfill -0.32\hfill & \hfill -0.08\hfill & \hfill 0.66\hfill & \hfill -0.16\hfill \\ {}\hfill 0.24\hfill & \hfill -0.24\hfill & \hfill -0.32\hfill & \hfill -0.18\hfill & \hfill -0.24\hfill & \hfill 0.74\hfill \end{array}\right]\left[\begin{array}{c}\hfill -9.5\hfill \\ {}\hfill -6\hfill \\ {}\hfill -20\hfill \\ {}\hfill 9.5\hfill \\ {}\hfill 7.5\hfill \\ {}\hfill -14\hfill \end{array}\right]=\left[\begin{array}{c}\hfill 8.29\hfill \\ {}\hfill -1.51\hfill \\ {}\hfill -1.91\hfill \\ {}\hfill 13.29\hfill \\ {}\hfill -6.11\hfill \\ {}\hfill -2.81\hfill \\ {}\hfill -9.21\hfill \\ {}\hfill 7.79\hfill \\ {}\hfill 13.39\hfill \\ {}\hfill -8.31\hfill \end{array}\right]\end{array} $$

The genotypic values calculated as the summation of the additive and dominance values are:

$$ \mathbf{g}=\mathbf{1}\upmu +{\mathbf{a}}_{\mathrm{h}}+{\mathbf{d}}_{\mathrm{h}}=\left[\begin{array}{c}\hfill 1\hfill \\ {}\hfill 1\hfill \\ {}\hfill 1\hfill \\ {}\hfill 1\hfill \\ {}\hfill 1\hfill \\ {}\hfill 1\hfill \\ {}\hfill 1\hfill \\ {}\hfill 1\hfill \\ {}\hfill 1\hfill \\ {}\hfill 1\hfill \end{array}\right](22.09)+\left[\begin{array}{c}\hfill -5.38\hfill \\ {}\hfill 9.42\hfill \\ {}\hfill -3.18\hfill \\ {}\hfill -0.38\hfill \\ {}\hfill 2.02\hfill \\ {}\hfill -4.28\hfill \\ {}\hfill -2.88\hfill \\ {}\hfill 3.12\hfill \\ {}\hfill 4.52\hfill \\ {}\hfill -1.78\hfill \end{array}\right]+\left[\begin{array}{c}\hfill 8.29\hfill \\ {}\hfill -1.51\hfill \\ {}\hfill -1.91\hfill \\ {}\hfill 13.29\hfill \\ {}\hfill -6.11\hfill \\ {}\hfill -2.81\hfill \\ {}\hfill -9.21\hfill \\ {}\hfill 7.79\hfill \\ {}\hfill 13.39\hfill \\ {}\hfill -8.31\hfill \end{array}\right]=\left[\begin{array}{c}\hfill 25\hfill \\ {}\hfill 30\hfill \\ {}\hfill 17\hfill \\ {}\hfill 35\hfill \\ {}\hfill 18\hfill \\ {}\hfill 15\hfill \\ {}\hfill 10\hfill \\ {}\hfill 33\hfill \\ {}\hfill 40\hfill \\ {}\hfill 12\hfill \end{array}\right]=\left[\begin{array}{c}\hfill {g}_{11}\hfill \\ {}\hfill {g}_{22}\hfill \\ {}\hfill {g}_{33}\hfill \\ {}\hfill {g}_{44}\hfill \\ {}\hfill {g}_{12}\hfill \\ {}\hfill {g}_{13}\hfill \\ {}\hfill {g}_{14}\hfill \\ {}\hfill {g}_{23}\hfill \\ {}\hfill {g}_{24}\hfill \\ {}\hfill {g}_{34}\hfill \end{array}\right]. $$

By comparing with the genotypic values in Table 2, the above result verifies that the multi-allelic partition of **g** = **1**μ + **a**_h_ + **d**_h_ = **1**μ + **W**_αh_**α**_h_ + **W**_δh_**δ**_h_ described by Eqs. 21–30 is correct. With the note that g_ij_ = g_ji_, a_ij_ = a_ji_ and d_ij_ = d_ji_, the genotypic variance (σ_g_^2^), additive variance (σ_a_^2^) and dominance variance (σ_d_^2^) are:

$$ \begin{array}{l}{\upsigma}_g^2={\displaystyle {\sum}_{\mathrm{i}=1}^{\mathrm{h}}{\displaystyle {\sum}_{\mathrm{j}=1}^{\mathrm{h}}{\mathrm{p}}_{\mathrm{i}}{\mathrm{p}}_{\mathrm{j}}{\mathrm{g}}_{\mathrm{i}\mathrm{j}}^2}}-{\upmu}^2=71.0419\\ {}{\upsigma}_{\mathrm{a}}^2={\displaystyle {\sum}_{\mathrm{i}=1}^{\mathrm{h}}{\displaystyle {\sum}_{\mathrm{j}=1}^{\mathrm{h}}{\mathrm{p}}_{\mathrm{i}}{\mathrm{p}}_{\mathrm{j}}{\mathrm{a}}_{\mathrm{i}\mathrm{j}}^2}}=20.1178\\ {}{\upsigma}_{\mathrm{d}}^2={\displaystyle {\sum}_{\mathrm{i}=1}^{\mathrm{h}}{\displaystyle {\sum}_{\mathrm{j}=1}^{\mathrm{h}}{\mathrm{p}}_{\mathrm{i}}{\mathrm{p}}_{\mathrm{j}}{\mathrm{d}}_{\mathrm{i}\mathrm{j}}^2}}=50.9241\end{array} $$

It is readily seen that σ_g_^2^ = σ_a_^2^ + σ_d_^2^.

### Mixed model and multi-allelic genomic relationship matrices

A mixed model to implement the multi-allelic haplotype model of Eq. 30 can be established with appropriate changes of matrix dimensions for **W**_αh_, **W**_δh_, **a**_h_, **d**_h_, **α**_h_ and **δ**_h_ in Eq. 30. A set of m SNP markers are assumed available, and r haplotype blocks of the m SNPs are defined across the genome. Haplotypes of all individuals are assumed known (e.g., constructed using a phasing or imputing software). Each haplotype block is treated as a ‘locus’ and each haplotype within a haplotype block is treated as an ‘allele’. The i_th_ haplotype block has h_i_ haplotypes, h_i_−1 additive effects, and n_δi_ dominance effects or heterozygous genotypes. Let n_α_ = total number of additive effects of all r haplotype blocks, n_δ_ = total number of dominance effects (or heterozygous genotypes) of all r haplotype blocks. Then, n_α_ = ∑_i = 1_^r^h_i_ − r, and n_δ_ = ∑_i = 1_^r^n_δi_. For a given sample of q individuals, the limit number of effects is 2q-1 for additive effects and is the number of heterozygous genotypes for dominance effects. For a sample with N observations on q individuals, the mixed model to implement the multi-allelic haplotype model of Eq. 30 can be expressed as:

31 $$ \mathbf{y}=\mathbf{X}\mathbf{b}+\mathbf{Z}\left({\mathbf{W}}_{\upalpha \mathrm{h}}{\boldsymbol{\upalpha}}_{\mathrm{h}}+{\mathbf{W}}_{\updelta \mathrm{h}}{\boldsymbol{\updelta}}_{\mathrm{h}}\right)+\mathbf{e} $$

where **Z** = N × q incidence matrix allocating phenotypic observations to each individual = identity matrix for one observation per individual (N = q), **α**_h_ = n_α_ × 1 column vector of haplotype additive effects, **W**_αh_ = q × n_α_ model matrix of **α**_h_, **δ**_h_ = n_δ_ × 1 column vector for dominance effects of haplotype genotypes, **W**_δh_ = q × n_δ_ model matrix of **δ**_h_, **α**_s_ = m × 1 column vector of single-SNP additive effects, b = c × 1 column vector of fixed effects such as heard-year-season in dairy cattle (c = number of fixed effects), and X = N × c model matrix of **b**. To define two equivalent models with complementary computing advantages and identical GBLUP and GREML results, the mixed model of Eq. 31 needs to be expressed as [8]:

32 $$ \mathbf{y}=\mathbf{X}\mathbf{b}+\mathbf{Z}\left({\mathbf{T}}_{\alpha \mathrm{h}}{\boldsymbol{\upalpha}}_{\mathrm{h}}+{\mathbf{T}}_{\updelta \mathrm{h}}{\boldsymbol{\updelta}}_{\mathrm{h}}\right)+\mathbf{e}=\mathbf{X}\mathbf{b}+\mathbf{Z}\left({\mathbf{a}}_{\mathrm{h}}+{\mathbf{d}}_{\mathrm{h}}\right)+\mathbf{e} $$

where **a**_h_ = **T**_αh_**α**_h_ = multi-allelic genomic breeding values, **d**_h_ = **T**_δh_**δ**_h_ = multi-allelic genomic dominance values, and each **T** matrix can be defined by any of the six definitions of genomic relationships we previously discussed and implemented [9]. For simplicity of notations, the **T** matrices are defined as: **T**_αh_ = **W**_αh_/k_αh_^1/2^, **T**_δh_ = **W**_δh_/k_δh_^1/2^, where k_αh_ = the average of diagonal elements of **W**_αh_**W**_αh_ ', and k_δh_ = the average of diagonal elements of **W**_δh_**W**_δh_ '. The genomic relationship matrices of Eq. 31 can thus be defined as:

33 $$ {\mathbf{A}}_{\mathrm{h}}={\mathbf{T}}_{\upalpha \mathrm{h}}{\mathbf{T}}_{\upalpha \mathrm{h}}\hbox{'} = \mathrm{multi}\hbox{-} \mathrm{allelic}\ \mathrm{genomic}\ \mathrm{additive}\ \mathrm{relationship}\ \mathrm{matrix} $$

34 $$ {\mathbf{D}}_{\mathrm{h}}={\mathbf{T}}_{\updelta \mathrm{h}}{\mathbf{T}}_{\updelta \mathrm{h}}\hbox{'} = \mathrm{multi}\hbox{-} \mathrm{allelic}\ \mathrm{genomic}\ \mathrm{dominance}\ \mathrm{relationship}\ \mathrm{matrix} $$

### Interpretation of multi-allelic and haplotype genomic relationship matrices

The multi-allelic genomic relationships of Eqs. 33 and 34 using multi-allelic markers such as microsatellite markers have the same interpretation and theoretical expectation as using SNP markers that are bi-allelic, e.g., a genomic additive relationship is expected to be twice the coancestry coefficient [8, 9]. Using either multi-allelic or bi-allelic markers under the assumption of no inbreeding, the theoretical expectation of genomic additive relationships is 0.5, 0.5, 0.25 and 0 for parent-offspring, full-sibs, half-sibs and unrelated individuals respectively, and the corresponding theoretical expectation of genomic dominance relationships is 0, 0.25, 0 and 0.

It is important to distinguish between single-locus multi-allelic markers such as microsatellite markers from haplotypes where each haplotype is treated as an ‘allele’ and each haplotype block is treated as a ‘locus’, because recombination between loci within a haplotype block generally exists, leading to lowered haplotype similarity than single-locus similarity among relatives. As the number of loci increases in each haplotype block, genomic relationships using haplotypes are expected to decrease from those using single-locus markers. Therefore, the utility of haplotype genomic relationships using Eqs. 33 and 34 is for genomic prediction using haplotypes, not for measuring relationships among individuals. The optimal block size and hence the number of haplotypes per block is an important issue for genomic prediction and could be determined by validation studies, as to be further discussed towards the end of this article.

### Two equivalent mixed models with complementary computing strategies

To establish mixed models using multi-allelic markers or haplotypes, assumptions for the first and second moments of the mixed model of Eq. 32 are: E(**y**) = **Xb**, E(**α**_h_) = E(**δ**_h_) = E(**α**_s_) = E(**δ**_s_) = 0, Var(**α**_h_) = σ_αh_^2^**I**_nα_, Var(**a**_h_) = **G**_αh_ = σ_αh_^2^**A**_h_**,** Var(**δ**_h_) = σ_δh_^2^**I**_nδ_, Var(**d**_h_) = **G**_δh_ = σ_δh_^2^**D**_h_, and Var(**e**) = **R** = σ_e_^2^**I**_N_, where σ_αh_^2^ = variance of multi-allelic additive effects, σ_δh_^2^ = variance of multi-allelic dominance effects, σ_e_^2^ = residual variance, and **I**_nα_, **I**_nδ_, **I**_m_ and **I**_N_ are identity matrices of orders n_α_, n_δ_, m and N, respectively. All random effects are assumed to be uncorrelated so that the phenotypic variance-covariance matrix is:

35 $$ \mathbf{V}=\mathrm{V}\mathrm{a}\mathrm{r}\left(\mathbf{y}\right)=\mathbf{Z}\left({\mathbf{G}}_{\upalpha \mathrm{h}}+{\mathbf{G}}_{\updelta \mathrm{h}}\right)\mathbf{Z}\hbox{'}+{\upsigma}_{\mathrm{e}}^2{\mathbf{I}}_{\mathrm{N}}=\mathbf{Z}\left({\upsigma}_{\upalpha \mathrm{h}}^2{\mathbf{A}}_{\mathrm{h}}+{\upsigma}_{\updelta \mathrm{h}}^2{\mathbf{D}}_{\mathrm{h}}\right)\mathbf{Z}\hbox{'}+{\upsigma}_{\mathrm{e}}^2{\mathbf{I}}_{\mathrm{N}} $$

To simply notations for the two equivalent mixed models, terms in Eqs. 32–35 are re-written as **α**_h_ = **τ**_1_, **δ**_h_ = **τ**_2_; **T**_αh_ = **T**_1_, **T**_δh_ = **T**_2_; **u**_i_ = **T**_i_**τ**_i_, i = 1,2; **A**_h_ = **S**_1_, **D**_h_ = **S**_2_; and σ_αh_^2^ = σ_1_^2^, σ_δh_^2^ = σ_2_^2^. Then, Eqs. 32 and 35 can be expressed as:

36 $$ \mathbf{y}=\mathbf{X}\mathbf{b}+\mathbf{Z}{\displaystyle {\sum}_{i=1}^2{\mathbf{T}}_{\mathrm{i}}{\boldsymbol{\uptau}}_{\mathrm{i}}}+\mathbf{e}=\mathbf{X}\mathbf{b}+\mathbf{Z}{\displaystyle {\sum}_{\mathrm{i}=1}^2{\mathbf{u}}_{\mathrm{i}}} $$

37 $$ \mathbf{V}=\mathrm{V}\mathrm{a}\mathrm{r}\left(\mathbf{y}\right)=\mathbf{Z}\left({\displaystyle {\sum}_{\mathrm{i}=1}^2{\upsigma}_{\mathrm{i}}^2{\mathbf{S}}_{\mathrm{i}}}\right)\mathbf{Z}\hbox{'}+{\upsigma}_{\mathrm{e}}^2{\mathbf{I}}_{\mathrm{N}}. $$

By defining **Z**_i_ = **ZT**_i_, an equivalent model of Eqs. 36 and 37 can be re-written as:

38 $$ \mathbf{y}=\mathbf{X}\mathbf{b}+{\displaystyle {\sum}_{\mathrm{i}=1}^2{\mathbf{Z}}_{\mathrm{i}}{\boldsymbol{\uptau}}_{\mathrm{i}}}+\mathbf{e} $$

39 $$ \mathbf{V}=\mathrm{V}\mathrm{a}\mathrm{r}\left(\mathbf{y}\right)={\displaystyle {\sum}_{\mathrm{i}=1}^2{\upsigma}_{\mathrm{i}}^2{\mathbf{Z}}_{\mathrm{i}}}{\mathbf{Z}}_{\mathrm{i}}\hbox{'}+{\upsigma}_{\mathrm{e}}^2{\mathbf{I}}_{\mathrm{N}}. $$

Equations 36 and 37 will be referred to as Model-I, and Eqs. 38 and 39 as Model-II. Model-I and Model-II are equivalent models because both models have identical E(**y**) and **V**, but these two models have different computational advantages that can be complementary to each other. For each model, two methods can be established for genomic prediction and estimation: the method of conditional expectation (CE) and the method of mixed model equations (MME), yielding a total of four methods for the two equivalent models. Model-I using CE is the best method for large numbers of SNP markers and multiple genetic factors, Model-II using MME is the best method for large numbers of individuals, and Model-I using MME and Model-II using CE have no computing advantage. Therefore, Model-I using CE and Model-II using MME will be used for genomic prediction and estimation. Using our previous naming of these two methods, GBLUP and GREML of Model-I using CE will be referred to as the CE set of formulations, and GBLUP and GREML of Model-II using MME as the QM set of formulation, where QM means ‘q > m’. These two methods yield identical results of prediction and estimation and are applicable to singular genomic relationship matrices. Assuming one observation per individual, CE based on Eqs. 36 and 37 is approximately easier to compute than QM based on Eqs. 38 and 39 if q < c + n_α_ + n_δ_ according to the size of the largest matrix to invert for each method (Table 3). Model-I using MME has no computing advantage over Model-I using CE due to the large coefficient matrix of MME and the requirement for full-rank relationship matrices; and Model-II using CE has no computing advantage over Model-I using CE due to the large **T** matrices to store in memory.

**Table 3:** Comparison of computational feasibility of four methods from the two equivalent models with haplotypes and SNPs for GBLUP and GREML

		Method of for calculating GBLUP	
		Conditional expectation (CE)	Mixed model equations (MME)
Model I, Eqs. 36 and 37	Largest matrix to invert	**V**, phenotypic variance-covariance matrix	**C**, coefficient matrix of MME
	Size of largest matrix to invert	q × q, assuming one observation per individual	c + 2q for **C**
	Largest matrix to store in memory	q × q **P** matrix	c + 2q for **C**
	Applicable to singular genomic relationship matrices	Yes, inverse relationship matrices avoided	No, inverse relationship matrices required
Model II, Eqs. 38 and 39	Largest matrix to invert	**V**, phenotypic variance-covariance matrix	**C**, coefficient matrix of MME
	Size of largest matrix to invert	q × q, assuming one observation per individual	c + n_α_ + n_δ_ for **C**
	Largest matrix to store in memory	q × n_α_ and q × n_δ_ **T** matrices, q × q **P** matrix	c + n_α_ + n_δ_ for **C**
	Applicable to singular genomic relationship matrices	Yes, inverse relationship matrices avoided	Yes, inverse relationship matrices avoided

### Genomic best linear unbiased prediction of genetic values (GBLUP)

Using the CE method of Model-I (Eqs. 36 and 37), GBLUP of the i_th_ type of genetic values for individuals in the training population is obtained as:

40 $$ {\widehat{\mathbf{u}}}_{\mathrm{i}}={\sigma}_{\mathrm{i}}^2{\mathbf{S}}_{\mathrm{i}}\mathbf{Z}\hbox{'}{\mathbf{V}}^{-1}\left(\mathbf{y}-\mathbf{X}\widehat{\mathbf{b}}\right)={\sigma}_{\mathrm{i}}^2{\mathbf{S}}_{\mathrm{i}}\mathbf{Z}\hbox{'}\mathbf{P}\mathbf{y}={\mathbf{S}}_{\mathrm{i}}{\boldsymbol{\upvarepsilon}}_{\mathrm{i}},\mathrm{i}=1,2 $$

where $\widehat{\mathbf{b}}={\left(\mathbf{X}\hbox{'}{\mathbf{V}}^{-1}\mathbf{X}\right)}^{-}\mathbf{X}\hbox{'}{\mathbf{V}}^{-1}\mathbf{y}$ = best linear unbiased estimator (BLUE) of fixed non-genetic effects, **P** = **V**^− 1^ − **V**^− 1^**X**(**X** ' **V**^− 1^**X**)^−^**X** ' **V**^− 1^, and ${\boldsymbol{\upvarepsilon}}_{\mathrm{i}}={\upsigma}_{\mathrm{i}}^2\mathbf{Z}\hbox{'}{\mathbf{V}}^{-1}\left(\mathbf{y}-\mathbf{X}\widehat{\mathbf{b}}\right)=\mathbf{Z}\hbox{'}\mathbf{P}\mathbf{y}=\mathrm{q}\times 1$ column vector of regressed phenotypic values of the training population as a regression of the i_th_ type of genetic values on the phenotypic values in the training population. Two equivalent methods with identical results can be used to predict genetic values of individuals without phenotypic observations (validation population): placing all individuals with or without records in the same mixed model by setting to zero the **Z** matrix for the validation population, or calculate predictions separately based on the regressed phenotypic values of the training population [8, 39]. Using this second method, GBLUP of the i_th_ type of genetic values for individuals in the validation population is calculated as:

41 $$ {\widehat{\mathbf{u}}}_{\mathrm{i}0}={\upsigma}_{\mathrm{i}}^2{\mathbf{S}}_{\mathrm{i}01}\mathbf{Z}\hbox{'}{\mathbf{V}}^{-1}\left(\mathbf{y}-\mathbf{X}\widehat{\mathbf{b}}\right)={\upsigma}_{\mathrm{i}}^2{\mathbf{S}}_{\mathrm{i}01}\mathbf{Z}\hbox{'}\mathbf{P}\mathbf{y}={\mathbf{S}}_{\mathrm{i}01}{\boldsymbol{\upvarepsilon}}_{\mathrm{i}} $$

where **S**_i01_ = q_0_ × q genomic relationship matrix between the training and validation populations for the i_th_ type of genetic values (q_0_ = number of individuals in the validation population).

Using the QM method (MME method of Model-II of Eqs. 38 and 39), genomic prediction first calculates the GBLUP of haplotype effects and then calculates GBLUP of genetic values. GBLUP of haplotype effects is obtained from solving the following MME:

42 $$ \left(\begin{array}{ll}\mathbf{X}\hbox{'}\mathbf{X}\hfill & \mathbf{X}\hbox{'}{\mathbf{Z}}_{\mathrm{g}}\hfill \\ {}{\mathbf{Z}}_{\mathrm{g}}\hbox{'}\mathbf{X}\hfill & {\mathbf{Z}}_{\mathrm{g}}\hbox{'}{\mathbf{Z}}_{\mathrm{g}}+\underset{\mathrm{i}=1}{\overset{2}{\oplus }}\left({\uplambda}_{\mathrm{i}}{\mathbf{I}}_{\mathrm{ti}}\right)\hfill \end{array}\right)\left(\begin{array}{c}\hfill \widehat{\mathbf{b}}\hfill \\ {}\hfill \widehat{\boldsymbol{\uptau}}\hfill \end{array}\right)=\left(\begin{array}{c}\hfill \mathbf{X}\hbox{'}\mathbf{y}\hfill \\ {}\hfill {\mathbf{Z}}_{\mathrm{g}}\hbox{'}\mathbf{y}\hfill \end{array}\right) $$

where $\widehat{\boldsymbol{\uptau}}=\left({\widehat{\boldsymbol{\uptau}}}_1,{\widehat{\boldsymbol{\uptau}}}_2\right)$, **Z**_g_ = (**Z**_1_, **Z**_2_), λ_i_ = σ_e_^2^/σ_i_^2^, t = n_α_, n_δ_, m and N for i = 1,2, respectively, and ⊕ denotes direct sum that defines a block diagonal matrix. With haplotype and SNP effects from Eq. 42, GBLUP of the i_th_ type of genetic values for individuals in the training and validation populations are obtained as:

43 $$ {\widehat{\mathbf{u}}}_{\mathrm{i}}={\mathbf{T}}_{\mathrm{i}}{\widehat{\boldsymbol{\uptau}}}_{\mathrm{i}} $$

44 $$ {\widehat{\mathbf{u}}}_{\mathrm{i}0}={\mathbf{T}}_{\mathrm{i}0}{\widehat{\boldsymbol{\uptau}}}_{\mathrm{i}} $$

where **T**_i0_ = the **T**_i_ matrix calculated using SNPs of the validation population. Equations 43 and 44 yield identical results as those of Eqs. 40 and 41. The prediction of total genotypic values in either training or validation population can be obtained from Eqs. 40 and 41 or 43 and 44 as: **ĝ** = ∑_i = 1_^2^**û**_i_ = predicted genotypic values of all individuals, and **ĝ**_0_ = ∑_i = 1_^2^**û**_i0_ = predicted genotypic values of the validation population. Prediction reliabilities of additive, dominance and genotypic predictions as the squared correlations between the genomic and true values has the same formulations as the R_ai_^2^, R_di_^2^ and R_gi_^2^ formulae in [8], and prediction accuracy is obtained as the square root of the reliability estimate.

### Genomic restricted maximum likelihood estimation (GREML) of variance components

Using the CE method of Model-I (Eqs. 36 and 37), the EM type GREML estimates of variance components are:

45 $$ {\upsigma_{\mathrm{i}}^2}^{\left(\mathrm{k}+1\right)}{{=\upsigma}_{\mathrm{i}}^2}^{\left(\mathrm{k}\right)}\mathbf{y}{\mathbf{P}}^{\left(\mathrm{k}\right)}\mathbf{Z}{\mathbf{S}}_{\mathrm{i}}\mathbf{Z}\hbox{'}{\mathbf{P}}^{\left(\mathrm{k}\right)}\mathbf{y}/\mathrm{t}\mathrm{r}\left({\mathbf{P}}^{\left(\mathrm{k}\right)}\mathbf{Z}{\mathbf{S}}_{\mathrm{i}}\mathbf{Z}\hbox{'}\right),\ \mathrm{i} = 1,2 $$

46 $$ {\upsigma_{\mathrm{e}}^2}^{\left(\mathrm{k}+1\right)}{{=\upsigma}_{\mathrm{e}}^2}^{\left(\mathrm{k}\right)}\mathbf{y}{\mathbf{P}}^{\left(\mathrm{k}\right)}{\mathbf{P}}^{\left(\mathrm{k}\right)}\mathbf{y}/\mathrm{t}\mathrm{r}\left({\mathbf{P}}^{\left(\mathrm{k}\right)}\right) $$

where k = iteration number. Using the QM method (Eqs. 38 and 39), the EM type GREML estimates of variance components are

47 $$ {\upsigma_{\mathrm{i}}^2}^{\left(\mathrm{k}+1\right)}={\widehat{\boldsymbol{\uptau}}}_{\mathrm{i}}^{\left(\mathrm{k}\right)}\hbox{'}{\widehat{\boldsymbol{\uptau}}}_{\mathrm{i}}^{\left(\mathrm{k}\right)}/\left[\mathrm{m}-\mathrm{t}\mathrm{r}\left({\mathbf{C}}^{\mathrm{i}\mathrm{i}\left(\mathrm{k}\right)}\right){\uplambda}_{\mathrm{i}}^{\left(\mathrm{k}\right)}\right] $$

48 $$ {\upsigma_{\mathrm{e}}^2}^{\left(\mathrm{k}+1\right)}={\widehat{\mathbf{e}}}^{\left(\mathrm{k}\right)}\hbox{'}{\widehat{\mathbf{e}}}^{\left(\mathrm{k}\right)}/\left\{\mathrm{N}-\left[\mathrm{r}-{\displaystyle {\sum}_{\mathrm{i}=1}^4\mathrm{t}\mathrm{r}\left({\mathbf{C}}^{\mathrm{i}\mathrm{i}\left(\mathrm{k}\right)}{\uplambda}_{\mathrm{i}}^{\left(\mathrm{k}\right)}\right)}\right]\right\} $$

where r is the rank of the coefficient matrix of Eq. 42, $\widehat{\mathbf{e}}=\mathbf{y}-\mathbf{X}\widehat{\mathbf{b}}-{\displaystyle {\sum}_{\mathrm{i}=1}^2{\mathbf{Z}}_{\mathrm{i}}{\widehat{\boldsymbol{\uptau}}}_{\mathrm{i}}}$, and **C**^ii^ is defined by:

$$ {\mathbf{H}}^{-1}={\left({\mathbf{Z}}_{\mathrm{g}}\hbox{'}\mathbf{M}{\mathbf{Z}}_{\mathrm{g}}+\underset{\mathrm{i}=1}{\overset{2}{\oplus }}{\uplambda}_{\mathrm{i}}{\mathbf{I}}_{\mathrm{ti}}\right)}^{-1}=\left[\begin{array}{cc}\hfill {\mathbf{C}}^{11}\hfill & \hfill {\mathbf{C}}^{12}\hfill \\ {}\hfill {\mathbf{C}}^{21}\hfill & \hfill {\mathbf{C}}^{22}\hfill \end{array}\right] $$

where **M** = **I**_N_ − **X**(**X** ' **X**)^−^**X** ', and ti = n_α_ for i = 1 and ti = n_δ_ for i = 2.

The EM-REML of Eqs. 45–48 are known to be slow but reliable to yield non-negative estimates of variance components. The AI-REML algorithm is fast but may be sensitive to starting values of variance components and may fail for extreme heritability levels. Formulations of AI-REML for the multi-allelic haplotype model in this article are straightforward extensions of the formulations we implemented for GVCBLUP [40].

### Integration of haplotype and single SNP effects in genomic prediction and estimation

Haplotype analysis and single SNP analysis can be analyzed jointly for genomic prediction in the same mixed model by adding single SNP effects from our previous work [8] to the mixed model of Eq. 31, i.e.,

49 $$ \mathbf{y}=\mathbf{X}\mathbf{b}+\mathbf{Z}\left({\mathbf{T}}_{\upalpha \mathrm{h}}{\boldsymbol{\upalpha}}_{\mathrm{h}}+{\mathbf{T}}_{\updelta \mathrm{h}}{\boldsymbol{\updelta}}_{\mathrm{h}}+{\mathbf{T}}_{\upalpha \mathrm{s}}{\boldsymbol{\upalpha}}_{\mathrm{s}}+{\mathbf{T}}_{\updelta \mathrm{s}}{\boldsymbol{\updelta}}_{\mathrm{s}}\right)+\mathbf{e} $$

50 $$ \mathbf{V}=\mathrm{V}\mathrm{a}\mathrm{r}\left(\mathbf{y}\right)=\mathbf{Z}\left({\upsigma}_{\upalpha \mathrm{h}}^2{\mathbf{A}}_{\mathrm{h}}+{\upsigma}_{\updelta \mathrm{h}}^2{\mathbf{D}}_{\mathrm{h}}+{\upsigma}_{\upalpha \mathrm{s}}^2{\mathbf{A}}_{\mathrm{s}}+{\upsigma}_{\updelta \mathrm{s}}^2{\mathbf{D}}_{\mathrm{s}}\right)\mathbf{Z}\hbox{'}+{\upsigma}_{\mathrm{e}}^2{\mathbf{I}}_{\mathrm{N}} $$

where **α**_s_ = m × 1 column vector of SNP additive effects, **T**_αs_ = q × m model matrix of **α**_s_, **δ**_s_ = m × 1 column vector of SNP dominance effects, **T**_δs_ = q × m model matrix of **δ**_s_, Var(**α**_s_) = σ_αs_^2^**I**_m_, Var(**a**_s_) = **G**_αs_ = σ_αs_^2^**A**_s_, Var(**δ**_s_) = σ_δs_^2^**I**_m_, Var(**d**_s_) = **G**_δs_ = σ_δs_^2^**D**_s_, **A**_s_ = genomic additive relationship matrix, and **D**_s_ = SNP genomic dominance relationship matrix, and where **A**_s_ = **T**_αs_**T**_αs_ ' and **D**_s_ = **T**_δs_**T**_δs_ '. Let **α**_*s*_ = **τ**_3_, **δ** = **τ**_4_; **u**_i_ = **T**_i_**τ**_i_, i = 1,…,4; **A**_s_ = **S**_3_, **D**_h_ = **S**_4_; and σ_αs_^2^ = σ_3_^2^, σ_δs_^2^ = σ_4_^2^. The GBLUP and GREML formulations to jointly include haplotype and single SNP additive and dominance effects essentially entails to extending the range of the subscript i from 2 to 4 for Eqs. 38–50.

GREML estimation using the joint mixed model with haplotype and SNP effects offer flexibility to estimate the heritability for various types of functional genomic information in any given autosome regions based on formulations we implemented in GVCBLUP [40], e.g., the additive and dominance heritabilities of haplotype blocks of all genes, all LD blocks, or all single SNPs. The heritability estimate for each type of genetic effects is: h_i_^2^ = σ_i_^2^/σ_y_^2^, where σ_y_^2^ = ∑_i = 1_^4^σ_i_^2^ + σ_e_^2^ = phenotypic variance. The total heritability of all types of genetic effects is the summation of all effect heritabilities, i.e., H^2^ = ∑_i = 1_^4^h_i_^2^. Genomic heritability estimation has flexibility unavailable from heritability estimation using pedigree relationships: the heritability estimation for a single SNP, a chromosome region, or a set of selected SNPs. Using the GREML formulae of Eqs. 35 and 36, the heritability for haplotype block j or SNP set j can be estimated as: ${\mathrm{h}}_{\mathrm{i}\mathrm{j}}^2=\left({\widehat{\boldsymbol{\uptau}}}_{\mathrm{i}\mathrm{j}}\hbox{'}{\widehat{\boldsymbol{\uptau}}}_{\mathrm{i}\mathrm{j}}/{\widehat{\boldsymbol{\uptau}}}_{\mathrm{i}}\hbox{'}{\widehat{\boldsymbol{\uptau}}}_{\mathrm{i}}\right){\mathrm{h}}_{\mathrm{i}}^2$, where ${\widehat{\boldsymbol{\uptau}}}_{\mathrm{ij}}$= subset j of ${\widehat{\boldsymbol{\uptau}}}_{\mathrm{i}}$, i = 1,…,4. Given sufficient computing power and sample sizes for extensive validation studies, these heritability estimates could help identify genomic regions and genes relevant to phenotypes within the framework of genomic prediction.

### Defining haplotype blocks using functional genomic information

**Fig. 1:**
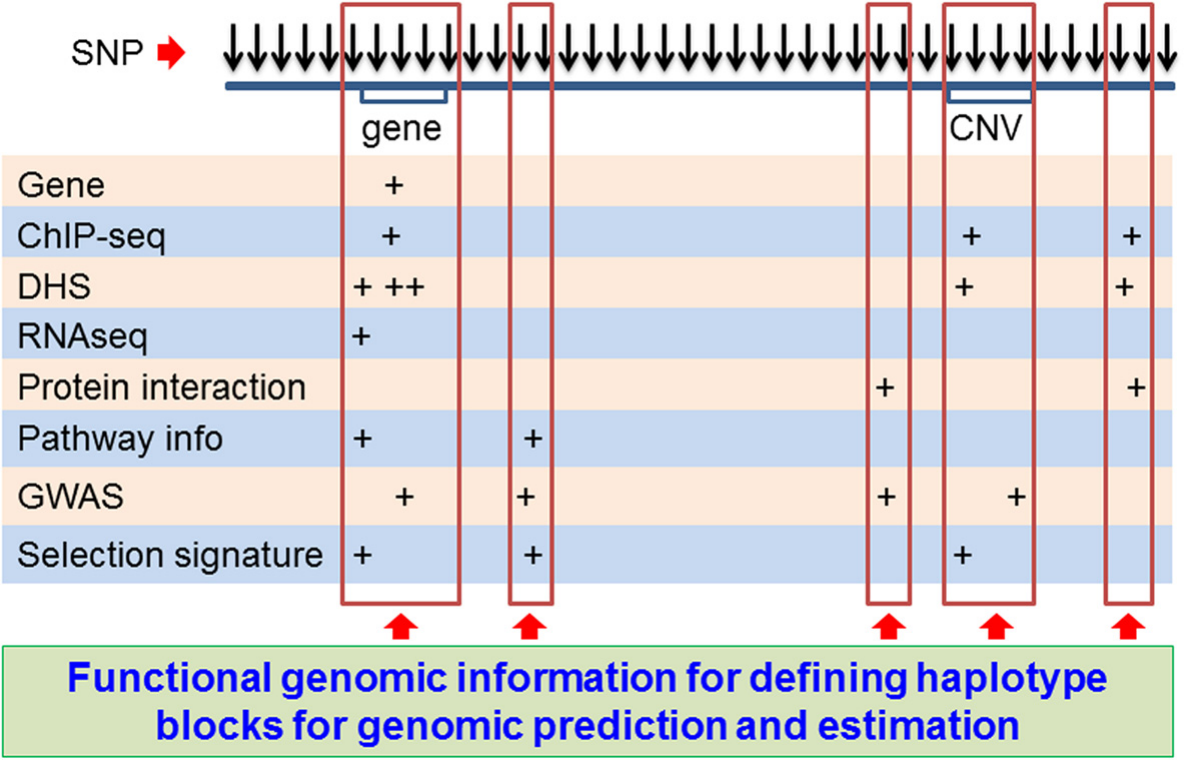
Integration of functional and structural genomic information for genomic selection. Haplotype blocks are defined using functional genomic information and are analyzed using the multi-allelic haplotype model in this article for genomic prediction and estimation. Single SNPs as structural genomic information can be used jointly with the haplotype analysis. (DHS = DNase I hypersensitive site)

The multi-allelic haplotype model can be used for the integration of functional genomic information with genomic prediction and estimation. This integration defines haplotype blocks using functional genomic information under the hypothesis that a chromosome region with functional information required more than a single point to affect a phenotype, followed by genomic prediction and estimation using a haplotype analysis such as the methods developed in this article. Each gene could be a ‘natural haplotype block’ and the use of gene blocks improved the prediction accuracy for some human phenotypes in our preliminary results [37]. Other types of functional information can also be used to define haplotype blocks, including ChIP-seq sites, DNA methylation sites, CNV, protein interaction, pathway information, GWAS results and selection signatures (Fig. 1). Other than ‘natural haplotype blocks’, the optimal block sizes for functional information with best prediction accuracy could be determined by extensive validation studies.

### Rare haplotypes, missing genotypic values

The mixed model approach outlined above allows rare haplotypes. In the extreme case of rare haplotypes with one observation per haplotype or haplotype frequency of 1/h when h is large, the multi-allelic model with the mixed model implementation still is applicable for additive effects and values. Missing genotypic values is a problem for dominance effects and values. The dominance effect defined by Eq. 9 requires the availability of all three genotypic values of a haplotype pair. Consequently, dominance effect is undefined with any missing genotypic value. We currently recommend ignoring any haplotype pair with missing genotypic value or values for defining dominance effects. For large haplotype blocks, nearly all individuals could be heterozygous so that such large blocks may not contribute to genomic prediction and estimation of dominance effects and values. This loss of dominance information should be a factor to consider in defining the block size.

## Conclusions

A multi-allelic haplotype model for genomic prediction and estimation is established using the quantitative genetics model that partitions a multi-allelic genotypic value into additive and dominance values, factorizes each additive value into a product between a function of allele frequencies and additive effect, and factorizes each dominance value into a product between a function of allele frequencies and dominance effect. Haplotype genomic additive and dominance relationship matrices and formulations are then derived for GBLUP and GREML utilizing haplotypes in haplotype blocks. These results fill a gap in the theory of quantitative genetics for multi-allelic genetic partition and provide a haplotype approach within the theory of quantitative genetics towards the integration of functional and structural genomic information for genomic selection.

## Availability of supporting data

The only data set used in this article is shown in Tables 1–2.

## Abbreviations

SNP: single nucleotide polymorphism
BLUP: best unbiased linear prediction
GBLUP: genomic BLUP
REML: restricted maximum likelihood estimation
GREML: genomic REML
EM: expectation-maximization
AI-REML: average information REML
CE: conditional expectation
MME: mixed model equations
